# Is robot-assisted pedicle screw placement really superior to conventional surgery? An overview of systematic reviews and meta-analyses

**DOI:** 10.1530/EOR-24-0062

**Published:** 2024-11-08

**Authors:** Wen-Xi Sun, Ming-Wang Qiu, Ze-hui Gao, Hong-Shen Wang, Bo-Lai Chen, Yong-Peng Lin

**Affiliations:** 1 The Second Clinical College of Guangzhou University of Chinese Medicine, Guangzhou, China; 2 Guangdong Provincial Hospital of Chinese Medicine, Guangzhou, China

**Keywords:** methodological quality, overview of reviews, pedicle screw placement, robot-assisted spine surgery

## Abstract

**Background:**

- Over the past two decades, modern spine surgery has become increasingly intellectualized and minimally invasive. However, whether using robots in spine surgery results in more accurate pedicle screw placement remains a topic of debate. This study aimed to evaluate the certainty and quality of the available evidence on the efficacy of robot-assisted pedicle screw placement.

**Methods:**

- We performed an overview of reviews including systematic reviews (SRs) and meta-analyses (MAs) regarding the accuracy of robot-assisted pedicle screw placement. Regarding the SRs/MAs, five electronic databases were searched from inception to 28 April 2023. There were no restrictions on the language or population. The quality and certainty of the evidence were evaluated with PRISMA, AMSTAR-2, ROBIS, Veritas plot, and GRADE tools.

**Results:**

- Fifteen SRs/MAs were analyzed. The findings indicated that the accuracy of pedicle screw placement in the robot-assisted group was not superior to that in the freehand group. All the SRs/MAs were of low or critically low quality. The main reasons for this include missing data, lack of transparency, lack of sensitivity analysis, and measurement of heterogeneity in the included studies, registration of reporting protocols, and deficiencies in the study inclusion methods and selection criteria.

**Conclusions:**

- While there is potential for robot-assisted pedicle screw placement to offer superior accuracy compared to conventional surgery, the current evidence is limited by methodological shortcomings. The quality of the studies analyzed was insufficient to provide a robust basis for developing clinical guidelines. Further high-quality research is necessary to confirm the benefits and establish clearer recommendations.

## Introduction

Various spinal disorders are among the major causes of disability worldwide, with a trend of persistently high morbidity rates contributing to the global healthcare burden (1, 2). Pedicle screw placement is a widely used technique in the treatment of severe degenerative spine diseases and spinal fractures, making it a fundamental procedure in spine surgery (3). Over the last two decades, modern spine surgery has become increasingly intellectualized and minimally invasive (4). Moreover, robot-assisted surgical technology is generally considered to offer greater accuracy and stability in procedures. Several recent studies and meta-analyses have highlighted the benefits of robot-assisted spine surgery for accurate pedicle screw placement (5, 6). However, other studies have found that patients who underwent robot-assisted pedicle screw placement had similar clinical efficacy and rates of complications as patients who underwent freehand pedicle screw placement (7, 8). Therefore, this study was conducted to evaluate the certainty and quality of the available evidence on the efficacy of robot-assisted pedicle screw placement.

## Data and methods

We conducted a registered overview of systematic reviews and meta-analyses of studies that evaluated the clinical efficacy of robot-assisted pedicle screw placement. This study followed the guidelines for systematic reviews stipulated in the Preferred Reporting Items for Systematic Reviews and Meta-Analyses (PRISMA) (9) and the Assessing the Methodological Quality of Systematic Reviews (AMSTAR-2) guidelines (10). The study protocol is available on PROSPERO (CRD42023422342).

### Inclusion and exclusion criteria

#### Inclusion criteria

These included: i) The systematic reviews (SRs)/meta-analyses (MAs) published in journals with no restrictions on the language or population; ii) patients who met the diagnostic criteria for spinal-related diseases, regardless of gender, age, ethnicity, region, and course of disease; and iii) the experimental group placing pedicle screws using any type of surgical robot, while the control group placed pedicle screws freehand.

#### Exclusion criteria

These included: i) Repeated published literature. ii) The patient did not meet the diagnostic criteria for spinal-related diseases. iii) Literature on non-surgical robot treatment of spinal-related diseases. iv) Studies with incomplete information or non-standard original text. v) Publications with only abstracts or translations, expert experience, animal experimental research, and other literature.

### Search strategy

A search for studies from inception to 28 April 2023, using Medline (via PubMed), OVID, Embase, Web of Science, and Cochrane Library databases. The search strategy of each database was structured to include terms related to ‘Robot-assisted surgery,’ ‘Pedicle screw,’ ‘Systematic review,’ and ‘Meta-analysis’ (Supplementary Material 1, see the section on supplementary materials given at the end of this article).

### Study selection

During the literature screening process, two evaluators (W-XS and M-WQ) initially reviewed the titles and abstracts of the retrieved studies. Irrelevant literature was excluded at this stage. The evaluators then assessed the remaining studies for inclusion by reading the full texts. Additionally, the reference lists of the selected literature were reviewed to identify any relevant literature. Any discrepancies between the evaluators were resolved through consensus meetings with the involvement of a third reviewer (Z-HG).

### Assessment of study quality

The investigators independently extracted and assessed data using three tools: AMSTAR-2, risk of bias in systematic reviews (ROBIS) (11), and PRISMA. Any discrepancies were resolved through discussions with a third reviewer (Z-HG). The outcomes were evaluated using the AMSTAR-2 tool, which is an internationally validated instrument for assessing the methodological quality of SRs. It contains 16 items with simple answers and a user guide. The overall score was based on weaknesses in seven critical domains: protocol registration, literature search, excluding studies, risk of bias (ROB) assessment, meta-analytical method, and publication bias.

The ROBIS tool was designed to critically assess the ROB in SRs in three phases: assessing relevance, identifying concerns regarding the review process, and judging the ROB. Phase II covers four domains through which bias may be introduced into an SR.

The PRISMA tool is an internationally validated instrument based on 27 items. Each item was evaluated as ‘yes,’ ‘partial yes,’ or ‘no’, representing full, partial, or no reports, respectively. The checklist included items deemed essential for the transparent reporting of an SR.

### Data extraction and analysis

Two researchers (M-WQ. and Z-HG) independently gathered the baseline data and outcome markers. Any disagreements were resolved by consultation with a third researcher (W-XS) or by seeking external advice. The data extraction content included basic information such as the first author, publication year, number of primary studies, type of primary studies, type of robots, and meta-analysis outcome measures; pedicle screw placement accuracy was the main outcome marker of interest. The GRADE tools were used to evaluate the overall strength of the evidence for pedicle screw placement accuracy (12).

The quality evaluation of the SRs/MAs adopted a method proposed by PANESAR named Veritas plots (13). The multiple evaluation items of the Veritas plots included the publication year, research type, AMSTAR-2, ROBIS, PRISMA, homogeneity, and publication bias. In the Veritas plots, the length represented the dimensional situation, and the coverage area represented the overall quality level of the included research literature. Four items were qualitatively evaluated: the publication year, research type, homogeneity, and publication bias. A quantitative evaluation was performed for the AMSTAR-2, ROBIS, and PRISMA. Over time, diseases and surgical robotics technology have been constantly changing, and the year of publication is also an important factor affecting the quality of articles (14, 15). If the research type of the included literature was a randomized controlled trial, its hierarchy of evidence was higher; if it contained a clinical cohort study (CCS) and other research literature, its hierarchy of evidence was lower. When more than half of the outcome indicators included in the literature had *P* ≥ 0.01, *I*² ≤ 50%, this indicated high homogeneity (16). If the literature used funnel plots or other methods to evaluate publication bias, the risk of publication bias was low. If the publication bias was ignored, the risk of publication bias was high (17). Each item in AMSTAR-2, which was standardized and used correctly, was given a score of 1 point. If not used or misused, a score of 0 points was assigned, with a maximum score of 11. The ROBIS scored 1 point for each item that was standardized and correctly used and 0 points for unused or misused items, with a maximum score of 25 points. Each item of the PRISMA was standardized and used correctly, with a score of 1 point. Incomplete use was scored with 0.5 points, and unused or misused items were scored with 0 points, with a total score of 27. The results of the included SRs/MAs were synthesized using a summary of the findings table, including relative and absolute results and the certainty of the evidence for each outcome.

### Principles for drawing the Veritas plots

The scores for each evaluation item were converted into rank numbers according to the method of processing medical statistical grade data (12). The total scores from the literature were used to evaluate the rank value.

## Results

### Selection and characteristics of the included studies

**Figure 1:**
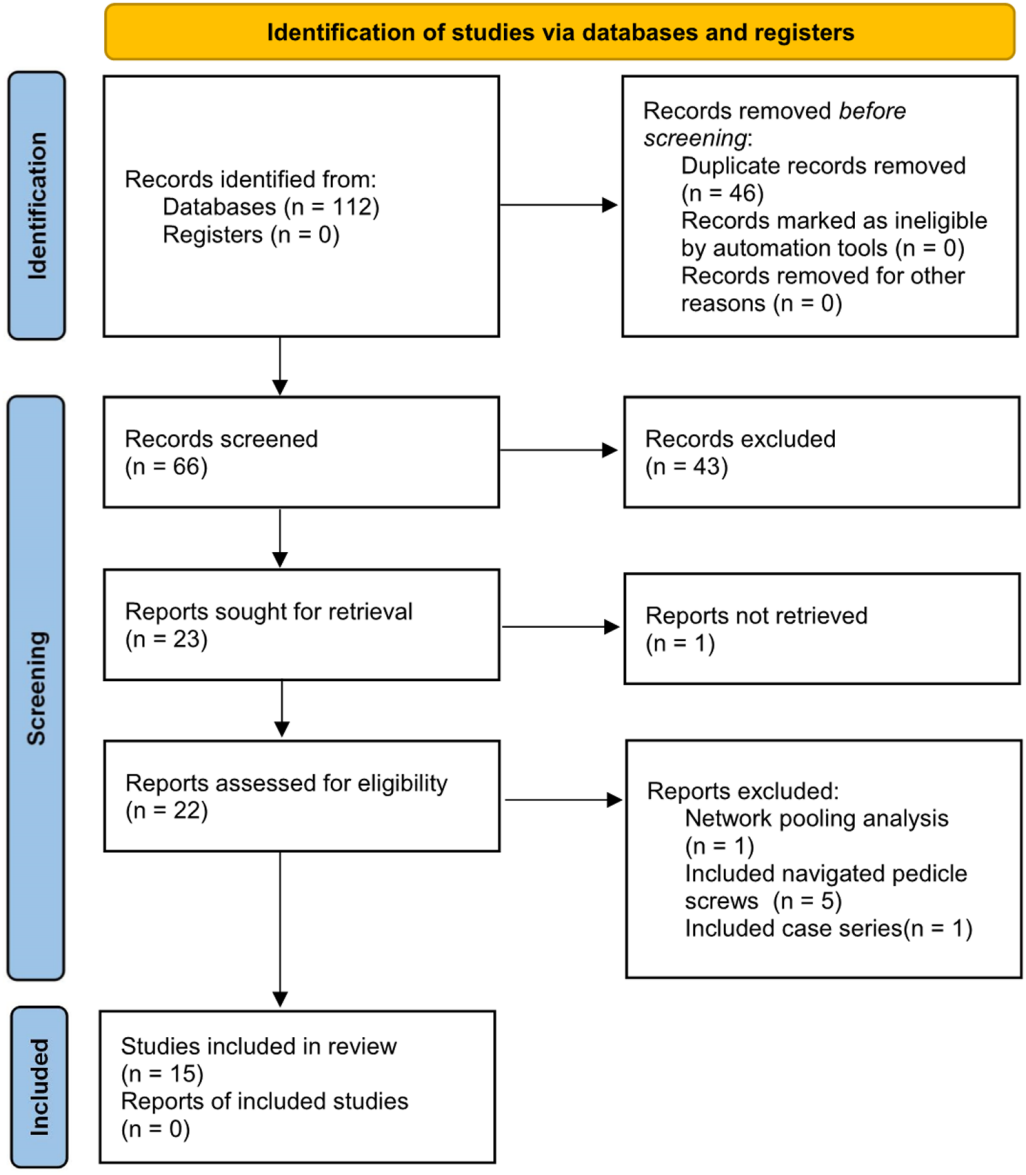
PRISMA flowchart for the selection of the studies.

Fifteen studies (6, 7, 18, 19, 20, 21, 22, 23, 24, 25, 26, 27, 28, 29, 30) were included in the final analysis. The numbers of included and excluded studies, with the reasons for exclusion, are shown in Fig. 1. All the SRs were published in English. The number of trials ranged from 6 to 76, and the sample size ranged from 606 to 5042. The details regarding the included articles and specific complications are presented in Table 1. Regarding the tools used to assess the ROB in these SRs, the Cochrane ROB was most often used in eight studies. This was followed by the Newcastle-Ottawa scale (NOS) and the Cochrane ROB used together in two studies. One study utilized the criteria set forth by the Oxford Centre for Evidence-Based Medicine (CEBM). The criteria set forth by both the CEBM and the Cochrane ROB were used for two of them, and three SRs did not provide information regarding the tools.

**Table 1:** Studies included in the systematic review and meta-analysis.

Reference	Year	Primary studies, *n*	Primary study type	Type of robots	Outcome measures
Tarawneh & Salem (27)	2021	7	RCT	Mazor/Tirobot	①, ②, ③,④, ⑤, ⑥, ⑧
Li *et al.* (18)	2020	9	RCT	Renaissance/Tirobot	①, ②, ③,④, ⑤, ⑥, ⑦, ⑨
Peng *et al.* (28)	2020	7	RCT	SpineAssist/Renaissance/TiRobot	①, ②, ④
Li *et al.* (25)	2021	13	RCT	Mazor/Tirobot	①, ④, ⑧, ⑨, ⑩
Zhou *et al.* (19)	2020	6	RCT/ PCS / RCS	Renaissance/Tirobot	①, ②, ③, ⑧, ⑨
Liu *et al.* (7)	2016	5	RCT/ PCS / RCS	NA	①
LuengoMatos *et al.* (20)	2022	10	RCT	SpineAssist/Renaissance/TiRobot	①, ②, ③, ④, ⑤, ⑦, ⑪, ⑫, ⑬, ⑭
Gao *et al.* (26)	2018	6	RCT	Mazor/Tirobot	①, ②, ③,④, ⑨
Fu *et al.* (29)	2021	15	RCT	NA	①, ②, ③, ⑤, ⑥, ⑦, ⑨, ⑫
Yu *et al.* (21)	2018	9	RCT/ RCS	Mazor/Rosa/NA	①, ②, ④, ⑩
Lopez *et al.* (30)	2023	76	PCS /RCS/ CCS	Mazor/Tirobot/Suzhou Zhuzheng robot/ SpineAssist/ Rosa/NA	①,③,④, ⑤, ⑩, ⑫, ⑬
Zhou *et al.* (22)	2023	7	RCT/ RCS /CS/ PCS	Mazor X/Tirobot/Cirq	①
Fatima *et al.* (6)	2021	19	RCT/ RCS / PCS	Mazor/Rosa/Tirobot	①, ②, ③, ④, ⑨, ⑩
Ghasem *et al.* (24)	2018	32	RCT/ RCS / PCS	SpineAssist/ Renaissance/Rosa/NA	①, ②, ⑤, ⑧, ⑩
Li *et al.* (23)	2020	23	RCT/ PCS / RCS	Excelsius GPS/Tirobot/ Rosa/SpineAssist/ Renaissance/NA	②, ③, ④, ⑤, ⑧, ⑩

CCS, case-control study; CS, case series; PCS, prospective cohort study; RCS, retrospective cohort study; RCT, randomized controlled trial.

Outcome measures: ① Pedicle screw placement accuracy; ② Surgical duration; ③ Radiation dose; ④ Fluoroscopy time; ⑤ Hospital stay (days); ⑥ VAS score; ⑦ ODI score; ⑧ Revision surgeries (immediate/delayed); ⑨ Proximal facet violations; ⑩ Complications; ⑪ Adverse events; ⑫ Intra-operative blood loss; ⑬ Total screw placement time; ⑭ EVA and SF-36.

## Results of the assessment of study quality

**Figure 2:**
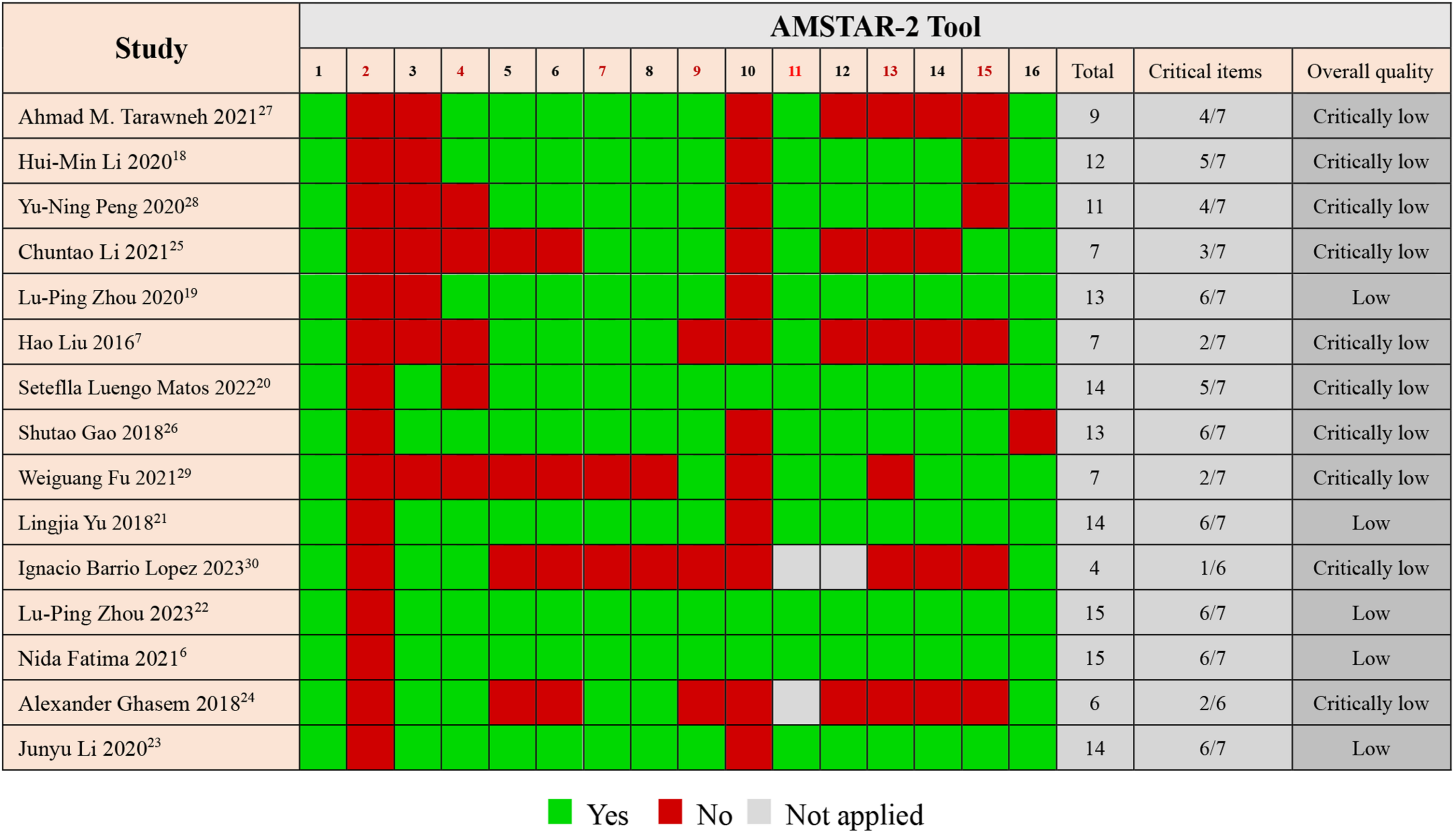
Evaluation of reviews according to the AMSTAR-2 tool.

According to AMSTAR-2, the results of overall quality showed five studies of low quality and nine of critically low quality. None of the studies were of high or moderate quality. The AMSTAR-2 results are illustrated in Fig. 2, with colored boxes for clarity. One SR had only one score on critical items, and five scored less than 50%. The three items with the lowest scores were the justification for the excluded studies (item 2), sources of funding for the included studies (item 10), and an explanation of the selection of the study designs for inclusion (item 3). The lack of these critical items was the reason for the low quality of the methodology.

**Figure 3:**
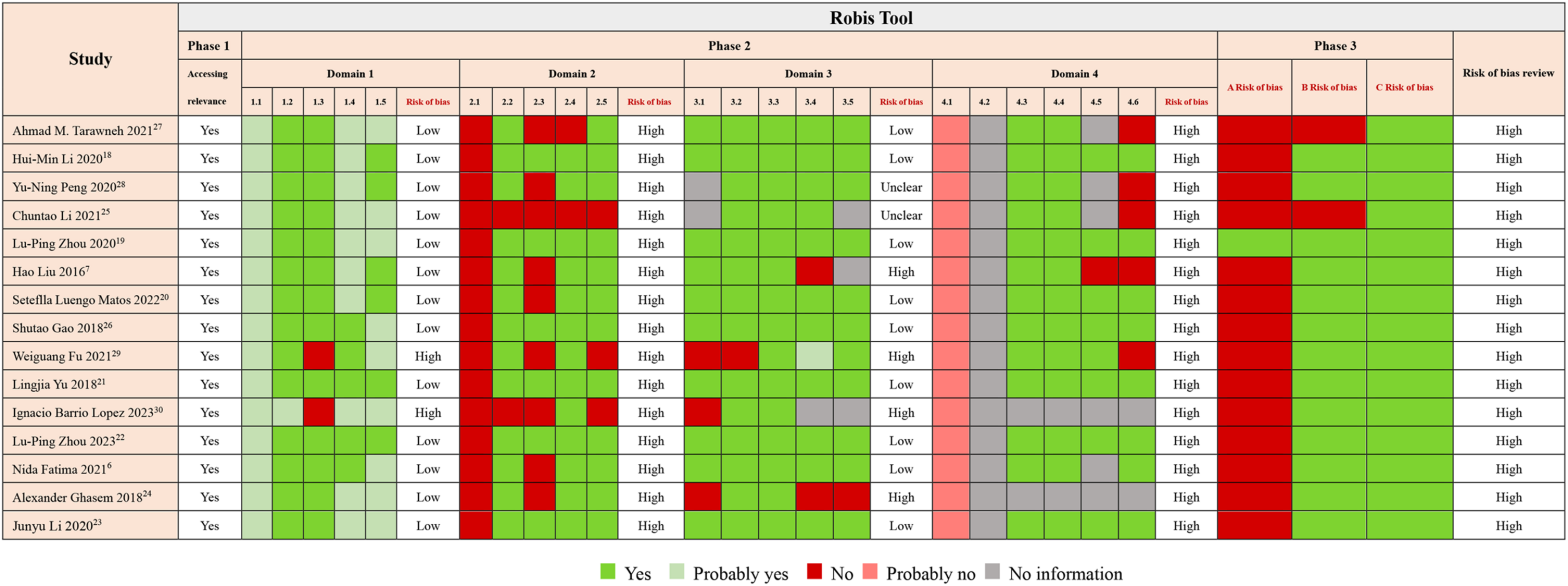
Evaluation of reviews according to the ROBIS tool.

Regarding the ROB assessment using the ROBIS (Fig. 3), the results found that all studies had a high ROB. Two studies had high ROBs for all domain items. In ROBIS phase 1, all the studies assessed relevance. There were four domains in ROBIS phase 2, including domain 1 (‘study eligibility criteria’), domain 2 (‘identification and selection of studies’), domain 3 (‘data collection and study appraisal’), and domain 4 (‘synthesis and findings’). Thirteen SRs/MAs were rated as having a low ROB in domain 1. Domains 2 and 4 were the worst scoring, with all studies rated as having a high ROB. Nine out of the fifteen SRs/MAs were rated as having a low ROB in domain 3. None of the studies disclosed plans or conducted protocol registration, and most did not fully or transparently present their systematic research.

Regarding the PRISMA tool, no SRs/MAs reported all tool items, but eight reported more than 80% (Supplementary material 2). No SRs/MAs reported any items regarding the abstracts. Projects with missing reports focused on methods and discussions such as presenting the full search strategies (item 7), synthesis methods (items 13a-e), reporting bias assessment (item 14), results of syntheses (item 20), risk of reporting biases in syntheses (item 21), and registration and protocol (item 24).

### Veritas plot evaluation results

**Figure 4:**
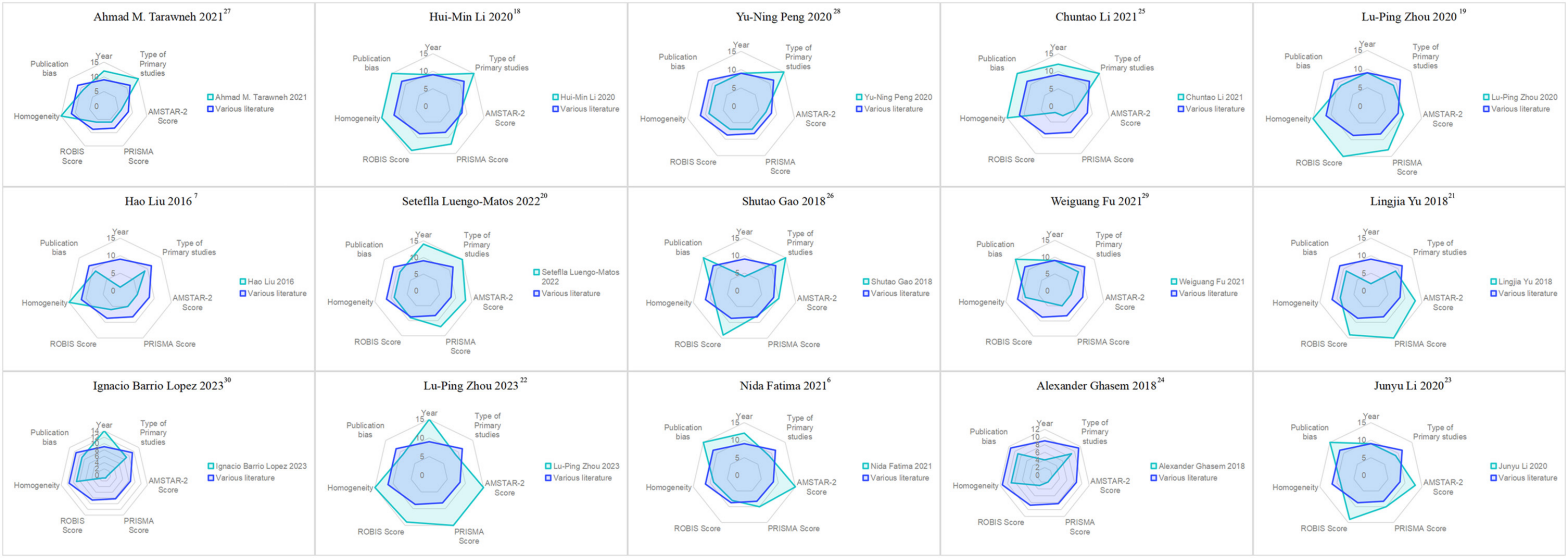
The Veritas plots regarding the 15 included reviews.

The results of the specific analysis are presented in Table 2. The Veritas plots of the 15 included reviews are shown in Fig. 4.

**Table 2:** Evaluation of reviews according to the veritas plots.

**Reference**	**Year**	**Primary study type**	**AMSTAR-2 Score**	**PRISMA score**	**ROBIS score**	**Homogeneity**	**Publication bias**	**Rank average score**
Tarawneh & Salem (27)	2021 (12)	RCT (15)	9 (6)	29 (6)	16 (6)	High (15)	None (9)	9.86
Li *et al.* (18)	2020 (9)	RCT (15)	12 (8)	34 (12)	21 (14)	High (15)	FP (15)	12.57
Peng *et al.* (28)	2020 (9)	RCT (15)	11 (7)	31 (7)	17 (7)	Low (9)	None (9)	9
Li *et al.* (25)	2021 (12)	RCT (15)	7 (5)	24 (3)	12 (2)	High (15)	FP (15)	9.57
Zhou *et al.* (19)	2020 (9)	RCT/PCS/RCS (9)	13 (10)	35 (13)	22 (15)	High (15)	None (9)	11.43
Liu *et al.* (7)	2016 (1)	RCT/PCS/RCS (9)	7 (5)	27 (5)	16 (6)	High (15)	None (9)	7.14
LuengoMatos *et al.* (20)	2022 (14)	RCT (15)	14 (13)	34 (12)	20 (9)	Low (9)	None (9)	11.57
Gao *et al.* (26)	2018 (4)	RCT (15)	13 (10)	32 (8)	21(14)	Low (9)	BT+ET (15)	10.71
Fu *et al.* (29)	2020 (9)	RCT (9)	7 (5)	27 (5)	15 (4)	Low (9)	FP+ET (15)	8
Yu *et al.* (21)	2017 (2)	RCT/RCS (9)	14 (13)	37 (15)	21 (14)	Low (9)	None (9)	10.14
Lopez *et al.* (30)	2022 (14)	RCT/PCS/ RCS/CCS (9)	4 (1)	15 (1)	10 (1)	N/A (9)	None (9)	6.29
Zhou *et al.* (22)	2023 (15)	RCT/ RCS /CS/ PCS (9)	15 (15)	37 (15)	21 (14)	High (15)	None (9)	13.14
Fatima *et al.* (6)	2021 (12)	RCT/RCS/PCS (9)	15 (15)	33 (10)	19 (8)	Low (9)	FP+ET (15)	11.14
Ghasem *et al.* (24)	2018 (4)	RCT/RCS/PCS (9)	6 (2)	17 (2)	13 (3)	N/A (9)	None (9)	5.43
Li *et al.* (23)	2020 (9)	RCT/PCS/ RCS (9)	14 (13)	33 (10)	21 (14)	Low (9)	HT +ET (15)	11.29

BT, Begg’s test; CCS: case–control study; CS: case series; ET, Egger’s test; FP, funnel plot; HT, Harbord’s test; PCS, prospective cohort study; RCS, retrospective cohort study; RCT: randomized controlled trial.

#### Visual analysis of graphics

Regarding the length of the Veritas plots, seven studies showed longer Veritas plots (6, 18, 19, 20, 21, 22, 23), indicating a better dimensional situation. Four studies (7, 24, 29, 30) had shorter Veritas plot image lengths, indicating poor dimensionality. The lengths of four studies (20, 22, 27, 28) were relatively average, indicating a small difference. Moreover, the lengths of three studies (19, 25, 26) were relatively uneven, indicating significant differences. Regarding the coverage area of the Veritas plots, four studies (18, 20, 22, 23) had a larger coverage area, indicating a higher overall level of literature. The coverage area of the three studies was relatively small (7, 24, 29), indicating a lower overall level of literature.

#### Comprehensive evaluation conclusion

A comprehensive analysis was conducted on the intuitive judgment of various research values on the Veritas plots and the average rank score. Li and Zhou *et al*. (19, 22) had the highest quality of literature in this study, with high and average scores in all dimensions. The most prominent defect in literature quality was noted in the study by Ghasem *et al*. (24), with low and uneven scores in most dimensions.

### Evidence synthesis

We assessed the critical outcomes of pedicle screw placement accuracy, and 12 MAs reported 18 critical outcomes (Table 3) (6, 7, 18, 19, 21, 22, 24, 25, 26, 27, 28, 29). Among them, seven indicators showed that the accuracy of robot-assisted pedicle screw placement was better than that of the freehand group. Nine indicators showed equal accuracy, and two showed lower accuracy in the robot-assisted group than in the freehand group. All the outcome indicators were of low or very low quality.

**Table 3:** Summary of findings regarding the critical outcomes of pedicle screw placement accuracy. Grade A and grade A + B positions are according to the Gertzbein–Robbins classification.

Outcomes/ primary study number	Screw numbers, *n*		Effect		Certainty (GRADE)	*P*
	Robot-assisted	Freehand	OR (95%CI)	RR (95% CI)		
Grade A						
7 RCTs	1298	1348	0.8422 (0.2238, 1.4605)		Very low (----)	<0.05
9 RCTs	1220	1256		1.05 (1.03 1.08)	Low (+---)	<0.05
7 RCTs	1220	1256	1.68 (0.82, 3.44)		Low (+---)	0.16
5 RCTs/PCS/RCS	606	499		1.08 (0.86 1.35)	Very low (----)	0.23
10 RCTs	NA			1.06 (1.01, 1.11)	Low (+---)	<0.05
6 RCTs	688	672		1.02(0.98, 1.06)	Low (+---)	0.35
15 RCTs	2748	3293	2.43(1.66, 3.54)		Low (+---)	<0.05
19 RCTs/PCS/RCS	3644	3695	1.68 (1.20, 2.35)		Low (+---)	<0.05
Grade A+B						
7 RCTs	1298	1348	0.8696 (0.2154, 1.5256)		Very low (----)	<0.05
7 RCTs	1220	1256	1.70 (0.47, 6.13)		Low (+---)	0.42
5 RCTs/PCS/RCS	606	499		1.02 (0.68 1.51)	Very low (----)	0.93
10 RCTs	NA			1.01 (1.00, 1.03)	Low (+---)	<0.05
6 RCTs	688	672		1.00 (0.97, 1.02)	Low (+---)	0.95
19 RCTs/PCS/RCS	3644	3695	1.454 (1.01, 2.37)		Low (+---)	0.05
Pedicle screw placement accuracy						
13 RCTs	2442	2285	2.91 (1.77 4.80)		Low (+---)	<0.05
6 RCTs/PCS/RCS	NA		0.24 (0.14 0.43)		Low (+---)	<0.05
9 RCTs/RCS	660	577	1.27 (0.73, 2.20)		Low (+---)	0.40
7 RCTs/RCS	697			0.880 (0.841, 0.914)	Very low (----)	0.07

CCS: case–control study; CS: case series; PCS: prospective cohort study; RCS: retrospective cohort study; RCT: randomized clinical trials.

## Discussion

As the quantity of clinical studies on robot-assisted pedicle screw placement rises, systematic reviews are required to support evidence-based clinical practice and the development of health policies. Evaluations of high-quality evidence can determine the appropriateness and safety of interventions, which in turn will support the development of guidelines and effectively inform policy development within healthcare systems (31). The overview of reviews is more informative and focused than the reviews themselves, with condensed evidence that aids in knowledge transfer and dissemination, as well as evidence used. In this study, we reviewed the previously published SRs/MAs of robotic-assisted pedicle screw placement to establish the validity and credibility of the evidence. Our objective was to offer clinicians more trustworthy and dependable evidence to inform therapeutic decision-making and the development of relevant policies and guidelines.

### Low quality of reportology in the evaluation of robot-assisted pedicle screw placement

The PRISMA statement results show that none of the studies included pre-registered protocol descriptions. In the SRs/MAs, selective reporting may result from failing to pre-register in accordance with the specifications due to the subjective descriptive analytic content. Item 8 of the PRISMA statement necessitates providing a search strategy for at least one database, and only one SR in this study met the requirements for a full report. As access to comprehensive and extensive literature is crucial for a well-written review, not reporting the search strategy would decrease the transparency and reproducibility of this study. Since there is a wide range of surgical robots and their technical aspects are complex, protocols for evaluating the effectiveness of robot-assisted pedicle screw placement are frequently oversimplified and inadequately documented.

### Low methodologic quality in the evaluation of robot-assisted pedicle screw placement

The results showed that all the SRs/MAs were of low or critically low methodological quality according to the AMSTAR-2 tool. This indicates that the results may not be entirely accurate and comprehensive. Introducing and registering a pre-program is an effective way to standardize the SRs/MAs process and improve transparency. Adherence to the pre-specified protocol can lower the risk of bias in the SRs/MAs process and ensure high-quality reporting of results. A thorough literature search strategy can effectively minimize the influence of publication bias on the findings. Furthermore, presenting a comprehensive list of screened articles and justifications for exclusions is a valuable approach to mitigate selective bias. Reporting of original research grant funding and related conflicts of interest aids readers in understanding whether financial sponsorship affects SRs/MAs outcomes. Although most of the SRs/MAs included used quality assessment tools to evaluate the risk of bias in the original studies, some did not investigate the potential impact of bias when the quality of the studies varied. This reduces the internal validity of the SRs/MAs results.

### High risk of bias in the evaluation of robot-assisted placing pedicle screws

The assessment using the ROBIS scale revealed that the evaluations of the SRs/MAs in this article were all assessed as high risk. Phase 2 demonstrated that the level of bias risk was high for both domain 2 and domain 4. The increased risk of bias in phase 3 was primarily influenced by the two aspects referenced in phase 2. In addition to searching normal databases, authors of SRs/MAs should also search at least MEDLINE, EMBASE, conference reports, clinical trial registry platforms, and references of included literature to guarantee complete data retrieval. In addition, authors conducting systematic evaluations ought to reduce the potential for error in assessing the risk of bias in primary studies. If primary studies are mistakenly deemed low-risk as opposed to high-risk, novel factors of confusion may be introduced. Appropriate handling of heterogeneity in studies, such as sensitivity analysis and subgroup analysis, can diminish the risk of bias during data synthesis. When significant differences exist in the characteristics of the two groups, authors of systematic evaluations should employ subgroup analysis to examine the effects of different interventions independently, even though dividing the subgroups tends to lessen the sample size and compromise statistical efficacy. Thus, it highlights the importance of conducting multicenter, high-quality RCTs with larger sample sizes for future studies.

### Robot-assisted pedicle screw placement may be superior to conventional surgery

According to the systematic evaluation conducted in this study, the majority of results suggest that robot-assisted pedicle screw placement is superior to conventional surgery. However, the results of the GRADE tool indicate that the majority of outcome indicators assessed were based on low-quality evidence (66.7%), with only a minor proportion of very low-quality evidence (33.3%). This suggests that the current systematic evaluation of outcome indicators for robotic-assisted pedicle screw implantation is of questionable quality, and the conclusions may lack credibility. Limitations were the most significant factor resulting in downgrading, primarily due to flaws in the methodological design of the primary studies. These flaws include incomplete implementation of blinding, failure to perform allocation concealment, and selective reporting, which can produce an additional risk of misleading results. Publication bias is a significant factor in the reduced quality of evidence. Most systematic evaluations included in this study suffered from incomplete retrieval, impacting the poor symmetry of the majority of systematic evaluation funnel plots. The quality of evidence obtained from systematic evaluations has been significantly impacted by the methodological limitations of the original studies. This can lead to biased results. To ensure the quality of future clinical randomized controlled trials, it is recommended that standard criteria from the CONSORT 2010 statement (32) are followed when controlling the quality of RCTs.

Hyun *et al*. (33) reported that robot-assisted surgery has a higher screw accuracy. However, Ringel *et al*. (34) found that the accuracy of the freehand group was superior to that of the robot-assisted group. Most studies indicated that robots could more continuously and accurately navigate and eliminate medical errors (e.g. screw misplacement) due to a surgeon’s inexperience and mental fatigue (35, 36, 37). Still, it remains unknown whether distinct robot types create different outcomes. The accuracy of freehand screw placement was highly dependent on the surgeon’s experience and awareness of intraoperative fluoroscopy outcomes (38). Experienced surgeons can insert screws more efficiently and safely.

Overall, this study demonstrates the need for methodological improvements, greater transparency, and methodological rigor regarding SRs/MAs for pedicle screw placement. This can lead to more reliable clinical conclusions. This study had certain limitations: i) Among the included studies, the methodological quality was generally low, and the conclusions were somewhat limited; ii) Due to the large variation in baseline data between the included studies, it was not possible to perform quantitative synthesis and analyze their effect values.

The overall methodological quality of published SRs/MAs on robot-assisted pedicle screw placement is far from satisfactory, and urgent improvements are needed to i) develop and register a protocol for SRs/MAs that provides justification for study design bias and selection; ii) conduct a comprehensive literature search; iii) provide a list of excluded studies with justification; iv) use appropriate statistical methods for MAs and a full discussion of heterogeneity; and v) report on the funding sources for the included primary studies. A concerted effort by policymakers, review authors, journal editors, and peer reviewers is required to achieve these goals.

## Conclusion

SRs/MAs are essential for writing guidelines. This study showed that the accuracy of robot-assisted pedicle screw placement may be superior to conventional surgery. SRs/MAs should be conducted better when using the necessary tools in the future. Therefore, there is still a need for high-quality, rigorously designed SRs/MAs for robot-assisted pedicle screw placement to obtain more objective and accurate medical evidence.

## Supplementary Materials

Supplementary Material 1

Supplementary Material 2

Supplementary Material 3

## ICMJE Conflict of Interest Statement

All the authors declare that there is no conflict of interest that could be perceived as prejudicing the impartiality of the study reported.

## Funding Statement

This work was partially supported by a grant from the National Natural Science Foundation of Chinahttp://dx.doi.org/10.13039/501100001809 (no. 82004385 to LYP), the Excellent Talents Program of Guangdong Provincial Hospital of Chinese Medicine (no. ZY2022YL17 to LYP), the Innovation Team Project of Guangdong Provincial Department of Education (no. 2021KCXTD020 to CBL), and the Scientific Research Project of Guangdong Provincial Hospital of Chinese Medicine (YN2023WSSQ05 to LYP).

## Author contribution statement

W-xS: Investigation, Data curation, Formal analysis, Methodology, Writing the original draft. M-wQ: Investigation, Methodology, Formal analysis. Z-hG: Investigation, Data curation. H-sW: Methodology, Resources. B-lC: Conceptualization, Methodology, Writing, reviewing & editing, Funding acquisition, Supervision. Y-pL: Conceptualization, Methodology, Writing, reviewing & editing, Funding acquisition, Supervision.
