# Supplementary material for: Is robot-assisted pedicle screw placement really superior to conventional surgery? An overview of systematic reviews and meta-analyses
Source: EFORT Open Rev. 2024 Nov 8;9(11):1077–86. doi: 10.1530/EOR-24-0062 (PMC11619727; doi:10.1530/EOR-24-0062)
Supplement: Supplementary Material 1 [file EOR-24-0062supplementary_material_1.pdf]

Prisma2020	Ahmad 2021
1 Identify the report as a systematic review.	YES
2 See the PRISMA 2020 for Abstracts checklist.	YES
3 Describe the rationale for the review in the context of existing knowledge.	YES
4 Provide an explicit statement of the objective(s) or question(s) the review addresses.	YES
5 Specify the inclusion and exclusion criteria for the review and how studies were grouped for the synt	YES
6 Specify all databases, registers, websites, organisations, reference lists and other sources searched or	YES
7 Present the full search strategies for all databases, registers and websites, including any filters and lin	NO
8 Specify the methods used to decide whether a study met the inclusion criteria of the review, includin	YES
9 Specify the methods used to collect data from reports, including how many reviewers collected data	YES
10a List and define all outcomes for which data were sought. Specify whether all results that were com	YES
10b List and define all other variables for which data were sought (e.g. participant and intervention ch	YES
11 Specify the methods used to assess risk of bias in the included studies, including details of the tool(	YES
12 Specify for each outcome the effect measure(s) (e.g. risk ratio, mean difference) used in the synthes	YES
13a Describe the processes used to decide which studies were eligible for each synthesis (e.g. tabulatin	YES
13b Describe any methods required to prepare the data for presentation or synthesis, such as handling	NO
13c Describe any methods used to tabulate or visually display results of individual studies and synthes	YES
13d Describe any methods used to synthesize results and provide a rationale for the choice(s). If meta-	YES
13e Describe any methods used to explore possible causes of heterogeneity among study results (e.g. s	NO
13f Describe any sensitivity analyses conducted to assess robustness of the synthesized results.	NO
14 Describe any methods used to assess risk of bias due to missing results in a synthesis (arising from	NO
15 Describe any methods used to assess certainty (or confidence) in the body of evidence for an outco	YES
16a Describe the results of the search and selection process, from the number of records identified in t	YES
16b Cite studies that might appear to meet the inclusion criteria, but which were excluded, and explain	YES
17 Cite each included study and present its characteristics.	YES
18 Present assessments of risk of bias for each included study.	YES
19 For all outcomes, present, for each study: (a) summary statistics for each group (where appropriate)	YES
20a For each synthesis, briefly summarise the characteristics and risk of bias among contributing studi	YES
20b Present results of all statistical syntheses conducted. If meta-analysis was done, present for each th	YES
20c Present results of all investigations of possible causes of heterogeneity among study results.	NO
20d Present results of all sensitivity analyses conducted to assess the robustness of the synthesized resu	NO
21 Present assessments of risk of bias due to missing results (arising from reporting biases) for each sy	NO
22 Present assessments of certainty (or confidence) in the body of evidence for each outcome assessed	NO
23a Provide a general interpretation of the results in the context of other evidence.	YES
23b Discuss any limitations of the evidence included in the review.	YES
23c Discuss any limitations of the review processes used.	YES
23d Discuss implications of the results for practice, policy, and future research.	YES
24a Provide registration information for the review, including register name and registration number, c	NO
24b Indicate where the review protocol can be accessed, or state that a protocol was not prepared.	NO
24c Describe and explain any amendments to information provided at registration or in the protocol.	NO
25 Describe sources of financial or non-financial support for the review, and the role of the funders or	YES
26 Declare any competing interests of review authors.	YES
27 Report which of the following are publicly available and where they can be found: template data co	NO

Li 2020	Peng 2020	Li 2021	Zhou 2020	Liu 2016	Setefilla 2022	Gao 2018
YES	YES	YES	YES	YES	YES	YES
YES	YES	YES	YES	YES	YES	YES
YES	YES	YES	YES	YES	YES	YES
YES	YES	YES	YES	YES	YES	YES
YES	YES	YES	YES	YES	YES	YES
YES	YES	YES	YES	YES	YES	YES
YES	NO	NO	YES	NO	NO	NO
YES	YES	NO	YES	YES	YES	YES
YES	YES	NO	YES	YES	YES	YES
YES	YES	NO	YES	YES	YES	YES
YES	YES	NO	YES	YES	YES	YES
YES	YES	NO	YES	NO	YES	YES
YES	YES	YES	YES	YES	YES	YES
YES	YES	YES	YES	YES	YES	YES
NO	NO	NO	NO	NO	NO	NO
YES	YES	YES	YES	YES	YES	YES
YES	YES	YES	YES	YES	YES	YES
YES	YES	NO	YES	NO	NO	YES
YES	NO	NO	NO	NO	NO	NO
YES	NO	YES	NO	YES	YES	YES
NO	NO	NO	YES	NO	YES	NO
YES	YES	YES	YES	YES	YES	YES
YES	YES	YES	YES	YES	YES	YES
YES	YES	YES	YES	YES	YES	YES
YES	YES	YES	YES	NO	YES	YES
YES	YES	YES	YES	YES	YES	YES
YES	YES	YES	YES	YES	YES	YES
YES	YES	YES	YES	YES	YES	YES
YES	YES	NO	YES	NO	YES	YES
YES	NO	NO	YES	NO	NO	YES
NO	NO	YES	NO	NO	YES	YES
NO	NO	NO	YES	NO	YES	NO
YES	YES	YES	YES	YES	YES	YES
NO	YES	NO	YES	YES	YES	YES
YES	YES	YES	YES	YES	YES	YES
YES	YES	YES	YES	YES	YES	YES
NO	NO	NO	NO	NO	NO	NO
NO	NO	NO	NO	NO	NO	NO
NO	NO	NO	NO	NO	NO	NO
YES	YES	NO	YES	YES	YES	NO
YES	YES	YES	YES	YES	YES	YES
YES	YES	YES	YES	NO	YES	NO

Fu 2021	YU 2018	Lopez 2023	Zhou 2023	Fatima 2021	Ghasem 2018	Li 2020
YES	YES	YES	NO	YES	NO	YES
YES	YES	YES	YES	YES	YES	YES
YES	YES	YES	YES	YES	YES	YES
YES	YES	YES	YES	YES	YES	YES
NO	YES	NO	YES	YES	YES	YES
YES	YES	YES	YES	YES	YES	YES
NO	YES	NO	YES	NO	NO	YES
NO	YES	NO	YES	YES	NO	YES
NO	YES	NO	YES	YES	NO	YES
YES	YES	YES	YES	YES	YES	YES
NO	YES	YES	YES	YES	YES	YES
NO	YES	NO	YES	YES	NO	YES
YES	YES	NO	YES	YES	NO	YES
YES	YES	YES	YES	YES	YES	YES
NO	NO	NO	NO	NO	NO	NO
YES	YES	YES	YES	YES	NO	YES
YES	YES	NO	YES	YES	NO	YES
YES	YES	NO	YES	YES	NO	YES
YES	YES	NO	YES	NO	NO	NO
YES	YES	NO	YES	YES	NO	YES
NO	YES	NO	YES	YES	NO	YES
YES	YES	YES	YES	YES	YES	YES
NO	YES	YES	YES	YES	YES	YES
YES	YES	YES	YES	YES	YES	YES
NO	YES	NO	YES	YES	NO	YES
YES	YES	NO	YES	YES	NO	YES
YES	YES	NO	YES	YES	NO	YES
YES	YES	NO	YES	YES	NO	YES
YES	YES	NO	YES	YES	NO	YES
YES	YES	NO	YES	NO	NO	NO
YES	YES	NO	YES	YES	NO	YES
NO	YES	NO	YES	YES	NO	NO
YES	YES	YES	YES	YES	YES	YES
NO	YES	NO	YES	YES	YES	YES
YES	YES	NO	YES	YES	YES	YES
YES	YES	YES	YES	YES	YES	YES
NO	NO	NO	NO	NO	NO	NO
NO	NO	NO	NO	NO	NO	NO
NO	NO	NO	NO	NO	NO	NO
YES	YES	NO	YES	NO	YES	YES
YES	YES	YES	YES	NO	YES	NO
YES	NO	NO	YES	YES	NO	NO
