# Supplementary material for: Is robot-assisted pedicle screw placement really superior to conventional surgery? An overview of systematic reviews and meta-analyses
Source: EFORT Open Rev. 2024 Nov 8;9(11):1077–86. doi: 10.1530/EOR-24-0062 (PMC11619727; doi:10.1530/EOR-24-0062)
Supplement: Supplementary Material 3 [file EOR-24-0062supplementary_material_3.pdf]

Medline (via Pubmed)

#1 MeSH Terms: "Robotic Surgical Procedures"

#2 MeSH Terms: "Pedicle Screws" OR "Vertebral Body"

#3 #1 AND #2

#4 Title/Abstract:"systematic review" OR "systematic reviews"

#5 Title/Abstract:"clinical trial overview" OR "clinical trial overviews"

#6 Title/Abstract:"meta-analysis" OR "meta-analyses" OR "meta analysis" OR "metaanalyses" OR "meta analyses" OR "metaanalysis"

#7 #4 OR #5 OR #6

#8 #1 AND #7

Embase

#1 'pedicle screw'/exp OR 'vertebra body'/exp

#2 'robot assisted surgery'/exp

#3 'systematic review'/exp AND 'meta analysis'/exp

#4 #1 AND #2 AND #3

Web of science

#1 (TS=(Robotic Surgical Procedures)) OR TS=(robot assisted surgery)

#2 (TS=(Pedicle Screws)) OR TS=(Pedicle Screw)

#3 (TS=(Vertebral Body)

#4 (#2 OR #3)

#5 (TS=(systematic review) OR TS=(systematic reviews) OR TS=(clinical trial overview) OR TS=(clinical trial overviews) OR TS=(meta-analysis) OR TS=(meta-analyses) OR TS=(meta analysis) OR TS=(meta analyses)

#6 (#1 AND #4 AND #5)

Cochrane library

#1 (robot assisted surgery):ti,ab,kw

#2 (Pedicle screw OR vetebra body):ti,ab,kw

#3 (systematic review OR meta analysis):ti,ab,kw

#4 #1 AND #2 AND #3

OVID

#1 (Robotic surgical procedures OR robot assisted surgery).mp. [mp=ti, ab, tx, ct, mc, st, or, tn, ps, ds, cb, rn, sq, mq, ge, tm, mi, bo, bt, hw]

#2 (pedicle screw OR vertebra body).mp. [mp=ti, ab, tx, ct, mc, st, or, tn, ps, ds, cb, rn, sq, mq, ge, tm, mi, bo, bt, hw]

#3 (meta analysis OR systematic review).mp. [mp=ti, ab, tx, ct, mc, st, or, tn, ps, ds, cb, rn, sq, mq, ge, tm, mi, bo, bt, hw]

#4 #1 AND #2 AND #3
